# Smoking Patterns among Primary School Students in Turkey

**DOI:** 10.1100/tsw.2006.288

**Published:** 2006-12-28

**Authors:** Yesim Uncu, Emel Irgil, Mehmet Karadag

**Affiliations:** ^1^ Department of Family Medicine, School of Medicine, University of Uludag, Bursa, Turkey; ^2^ Department of Public Health, School of Medicine, University of Uludag, Bursa, Turkey; ^3^ Department of Chest Disease, School of Medicine, University of Uludag, Bursa, Turkey

**Keywords:** smoking, primary school children, prevention, healthy cities, Turkey

## Abstract

Cigarette smoking continues to be a threat to global health. The number of cigarettes smoked per person tends to increase each year, and the age of starting seems to be dropping. The research related to cigarette smoking conducted among young people generally studied high school or university students. However, studies have shown that students usually start smoking during the primary school period out of curiosity or imitation. The purpose of the present study was to find the prevalence of cigarette smoking among primary school students and the reasons for starting smoking, and to determine the characteristics of cigarette smoking of their parents. This study was conducted among 17 primary schools chosen according to their socioeconomic situations in different municipality districts in Turkey, with 9,408 students participating. Data were obtained by questionnaire. The mean age to start smoking was 11.7 ± 1.6; 82.9% of the students who took part in this study had never smoked before, 13.4% had tried smoking at least once, and 3.7% had been smoking regularly. The biggest reason for smoking was just curiosity or imitation. It was determined that a risk factor for students to start smoking was parents who smoke. The 17% smoking rate among primary school students was high in our opinion and prevention studies initiated. In addition, the effects of cigarette-smoking parents on students who start smoking should also be considered.

## INTRODUCTION

Smoking stands out as one of the leading causes of early death and is a preventable disease. Each year, 5 million people die due to smoke-related reasons; most of these deaths are in developing countries[1]. While tobacco use has decreased in developed countries such as the U.S., Japan, and the U.K. since the 1960s, smoking in Turkey has increased and the age at which people start to smoke is still dropping, according to several studies[2,3,4,5,6]. Turkey has a population of 70 million people and almost half of this population (total prevalence: 43.6%) are smokers[7]. Studies that include adolescents have shown that 25.2% of males and 10.5% of females smoke[8]. However, the studies focused on youth generally include high school and university students, but do not include primary school children[9]. Yet, Fidler et al.[10] have shown that a child who tries a cigarette once at the age of 11 years develops a greater risk of being an active smoker during adolescence.

Bursa is situated in the midwestern part of Turkey and is the fourth largest city in Turkey, with a population of 2,106,687 persons. Bursa was the first city from Turkey to join the “Healthy Cities Project” (founded by WHO) on July 5, 2000. This initiative is a long-term development project whose aim is to improve people’s physical, psychological, social, and environmental welfare under the principles of the WHO’s “Health for All in the 21st Century”, “Declaration of Human Rights”, and “Local Agenda 21”.

The present study investigated the frequency of tobacco use, the reasons for starting smoking, and the factors related to smoking in primary school children.

## METHODS

The Healthy Cities Project is an ongoing initiative carried out by Bursa’s stakeholders within context of Local Agenda 21. As the health education group, Uludag University, Provincial Directorate of Health, Provincial Directorate of Education, and Bursa Metropolitan Municipality decided to initiate an investigation via questionnaires to determine smoke-related concerns in primary school children.

Seventeen randomized primary schools, chosen according to their socioeconomic status from different municipality districts in Bursa, were included in the study. A questionnaire was distributed to the 9,408 children attending classes between 5th and 8th grade.

The questionnaire was prepared by Uludag University faculty staff and was distributed after being approved by the Ethical Committee of the Directorate of National Education. The parents of the children were informed, and their consents were taken. Data on students, such as age, gender, smoking habits, presence of smoking people in their family, and their reasons for beginning to smoke, were obtained from those questionnaires. Questionnaires were anonymous in order to encourage the children to give true answers.

### Statistical Methods

The data of the study were analyzed by using the Statistical Package for the Social Sciences (SPSS, Chicago, IL, version 11.0). Differences in proportions for categorical variables were tested by chi-square. The data were expressed as means ± SD. A $p$ value of less than 0.05 was considered significant.

## RESULTS

There were 9,408 children out of 12,171 (77.3%), aged between 11 and 16, attending classes in 17 schools within our study group, who were contacted and included in the investigation. The mean age of the children was 12.9 ± 1.2 (males, 13.0 ± 1.2; females, 12.8 ± 1.2). The age and gender distribution of our study group is shown in Table 1.

The smoking habits of the children were revealed to be as follows: 82.9% had never smoked, 13.4% had tried smoking at least once, and 3.7% had smoked previously and quit or were still smoking. The smoking rate was 6.2% among males and 1.0% among females (Table 2). The mean age for starting to smoke was 11.7 ± 1.6 (varying between 7 and 16 years old). Whereas the mean starting age for males was 11.7 ± 1.7, the mean starting age was 12.0 ± 1.4 for females. It was determined that the older the children were, the higher the rate of smoking (Fig. 1). When the smoking habits of the parents of the children were examined, it was determined that families of 6,623 (70.4%) children had at least one member who smoked. The correlation between the presence of a parent who smoked and a child who smoked was significant (c2 = 8.81, $p < 0.05$) (Table 3). Furthermore, it was found that children mostly smoke cigarettes when they are with their friends (67.8%). Whereas the most common reason to start smoking was imitating grown-ups (31.9%), the second leading reason was mere curiosity (30.2%). One other interesting result of the study was the answer that children gave (50.4%) when asked how they obtain cigarettes. They replied, “We buy it” (Table 4).

**TABLE 1**
**Distribution of the Study Group in Terms of Age and Gender**

| Age | Gender | | | | | |
|---|---|---|---|---|---|---|
| | Male | | Female | | Total | |
| | n | % | n | % | n | % |
| 11 | 694 | 14.0 | 708 | 15.9 | 1,402 | 14.9 |
| 12 | 1,139 | 23.0 | 1,105 | 24.9 | 2,244 | 23.9 |
| 13 | 1,398 | 28.2 | 1,219 | 27.4 | 2,617 | 27.8 |
| 14 | 1,178 | 23.8 | 1,146 | 25.7 | 2,324 | 24.7 |
| 15 | 457 | 9.2 | 250 | 5.6 | 707 | 7.5 |
| 16 | 90 | 1.8 | 24 | 0.5 | 114 | 1.2 |
| Total* | 4,956 | 52.7 | 4,452 | 47.3 | 9,408 | 100.0 |

*Row percentage.

**TABLE 2**
**Smoking Habits of Children in Terms of Gender**

| Smoking Status of Students | Gender | | | | | |
|---|---|---|---|---|---|---|
| | Male | | Female | | Total | |
| | n | % | n | % | n | % |
| Nonsmoker | 3,702 | 74.7 | 4,096 | 92.0 | 7,798 | 82.9 |
| Experimental smoker | 946 | 19.1 | 313 | 7.0 | 1,259 | 13.4 |
| Irregular smoker | 213 | 4.3 | 30 | 0.7 | 243 | 2.6 |
| Regular smoker | 84 | 1.7 | 10 | 0.2 | 94 | 1.0 |
| Exsmoker | 11 | 0.2 | 3 | 0.1 | 14 | 0.1 |
| Total | 4,956 | 100.0 | 4,452 | 100.0 | 9,408 | 100.0 |

$\chi^2 = 179.9$; $p < 0.0001$.

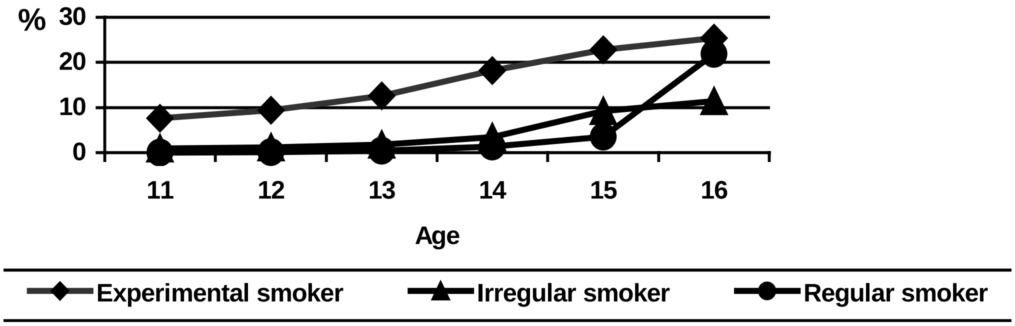

**FIGURE 1.** Percentage distribution of the frequency for cigarette smoking with regard to age.

**TABLE 3**
**Relationship Between Children Who Smoke and Presence of a Smoking Parent**

| Presence of a Smoking Parent | Smoker | | Nonsmoker | | Total | |
|---|---|---|---|---|---|---|
| | n | % | n | % | n | % |
| Yes | 272 | 77.5 | 6351 | 70.1 | 6623 | 70.4 |
| No | 79 | 22.5 | 2706 | 29.9 | 2785 | 29.6 |
| Total* | 351 | 3.7 | 9057 | 96.3 | 9408 | 100.0 |

*Row percentage; $\chi^2 = 8.81$; $p < 0.05$.

**TABLE 4**
**Characteristics of Children Who Smoke**

| Characteristics | Gender | | | | | |
|---|---|---|---|---|---|---|
| | Male | | Female | | Total | |
| | n | % | n | % | n | % |
| When do they smoke? | | | | | | |
| When together with friends | 212 | 68.8 | 26 | 60.5 | 238 | 67.8 |
| When alone | 71 | 23.1 | 13 | 30.2 | 84 | 23.9 |
| Always | 25 | 8.1 | 4 | 9.3 | 29 | 8.3 |
| Where do they get cigarettes? | | | | | | |
| Home | 31 | 10.1 | 11 | 25.6 | 42 | 12.0 |
| Friends | 117 | 38.0 | 15 | 34.9 | 132 | 37.6 |
| Buy at store | 160 | 51.9 | 17 | 39.5 | 177 | 50.4 |
| Reasons for starting | | | | | | |
| Imitating grown-ups | 101 | 32.8 | 11 | 25.6 | 112 | 31.9 |
| Curiosity | 95 | 30.8 | 11 | 25.6 | 106 | 30.2 |
| To prove him/herself | 32 | 10.4 | 8 | 18.6 | 40 | 11.4 |
| Anxiety, stress | 37 | 12.0 | 11 | 25.6 | 48 | 13.7 |
| Unable to break up friends | 39 | 12.7 | 2 | 4.6 | 41 | 11.7 |
| All | 4 | 1.3 | — | 0.0 | 4 | 1.1 |

In the last section, the children's thoughts on quitting smoking were investigated. Whereas 61.8% of smokers said that they considered quitting, 10% stated that they were uncertain. They remarked that the most important factor that might be effective in quitting was to see that it destroys their health (40.5%, n = 142) (see Table 5).

## DISCUSSION

In the present study, we found the prevalence of smoking in 11–16 year olds to be 3.7%. When this value is compared to the other results reported in the literature, this figure may be evaluated as low. Ogel et al.[9] conducted an investigation comprising nine provinces of Turkey including children between the ages of 10

**TABLE 5**
**Thoughts of Children on Quitting Smoking**

| | Male | | Female | | Total | |
|---|---|---|---|---|---|---|
| | n | % | n | % | n | % |
| Does he/she want to quit smoking? | | | | | | |
| Yes | 194 | 63.0 | 23 | 53.5 | 217 | 61.8 |
| Maybe | 32 | 10.4 | 3 | 7.0 | 35 | 10.0 |
| Doesn't know | 48 | 15.6 | 12 | 27.9 | 60 | 17.1 |
| Doesn't want to | 34 | 11.0 | 5 | 11.6 | 39 | 11.1 |
| An effective factor for quitting? | | | | | | |
| To see that it harms his/her own health | 122 | 39.6 | 20 | 46.5 | 142 | 40.5 |
| Education about the harms of smoking | 79 | 25.6 | 11 | 25.6 | 90 | 25.6 |
| To see it harming other people's health | 50 | 16.3 | 6 | 14.0 | 56 | 16.0 |
| Increases in cigarette prices | 55 | 17.9 | 5 | 11.6 | 60 | 17.1 |
| All | 2 | 0.6 | 1 | 2.3 | 3 | 0.8 |

and 12 years, and revealed that the rate of primary school children who smoked tobacco at least once in their lifetime was 16.1%. According to the results of a study conducted by the WHO, the CDC, and the Canadian Public Health Association, between 1999–2005 across 132 countries in children aged 13–15 years, the rate of smoking in youth was approximately 8.9%. This rate was reported as 17.9% in Europe, 17.5% in the U.S., 6.5% in the West Pacific, 5.0% in the East Mediterranean, and 4.3% in Southeast Asia[1]. The low rate of smoking observed in our study was partially due to the relatively young age profile of our study group. When children below the age of 13 were excluded, this rate increased to 5.1%, which is consistent with the value reported for Mediterranean countries.

The significant point here is the high rate of children who have tried smoking at least once in their lives. When we add the children who have tried smoking at least once in their lifetimes, the smoking ratio is raised to 17.1%. Another impressive result is that 19.9% of students who smoke began this habit before age of 11 years. Fidler et al.[10] have underlined the importance of this point in their investigation of a group consisting of children aged 11 years. They showed that in this age group, the rate of children who tried smoking at least once was 14% and those children had a 2.1 times greater risk of smoking even after 3 years compared to those who had never tried smoking. The researchers labeled this condition the "sleeper effect"[10]. In other prospective studies, this sensitivity was shown as well[11,12,13].

When the smoking rates of children were examined in terms of gender difference, males were found to be smoking more than females, conforming with the same condition in adults. Ogel et al.[9], in their study, found that male children smoked two times more than females. However, according to the results of Global Youth Tobacco Survey (GYTS), no gender difference was found in the U.S., the East Mediterranean, and Europe. But studies conducted in developing countries, such as South Africa and Indonesia, have shown that smoking is seen more commonly in males compared to females[14,15,16].

One of the most enlightening results of this study was the effect of environment on children smoking during those ages. As shown in the previous studies, the smoking habits of a family bear considerable correlation with the smoking habits of a child[14,17,18,19,20]. Bauman et al.[21] showed in their studies that the risk of smoking for adolescents between the ages 12 and 14 years with smoking parents was twice greater compared to the children whose families did not smoke. When the reasons underlying smoking were investigated in the study group, most of the children said that they began smoking because they wanted to imitate adults, because they were curious, or because they wanted to prove themselves to others. They usually got their cigarettes through their friends or bought them from shops.

Osaki et al.[2] have reported that in Japan, even children with school uniforms do not encounter any difficulties while buying cigarettes. However, it is illegal in Japan, as in Turkey, to sell cigarettes to individuals under the age of 18 years. Thus, the most important thing seems to be not passing laws, but making them applicable[2]. An introverted personality was found to be a factor in increasing addictions in studies investigating the leading factors contributing to smoking by adolescents[22], but personality characteristics were not researched in our study.

The study had some limitations. Since the study was realized throughout the schools, it included only children attending school, thus excluding children not attending school. However, since the length of mandatory education was increased to 8 years, the rate of children attending primary schools is as high as 97.6%; thus, we can say that those results reflect the overall status of children between 11 and 16 years in Bursa. Another limitation originated from the questionnaire-based character of the study. In such studies, children may show their smoking habits more or less. However, previous studies of smoking habits based on questionnaires have shown this method to be reliable[23]. Moreover, it was encouraging for children to give true answers when excluding their names in the questionnaire.

## ACKNOWLEDGMENTS

This study was presented on the 15th of July at the 13th World Conference on Tobacco OR Health in Washington, D.C. The authors would like to thank the Healthy City Project Unit of Bursa.
